# Measuring Maternal Mortality: Three Case Studies Using Verbal Autopsy with Different Platforms

**DOI:** 10.1371/journal.pone.0135062

**Published:** 2015-08-21

**Authors:** Siân L. Curtis, Robert G. Mswia, Emily H. Weaver

**Affiliations:** 1 Department of Maternal & Child Health, Gillings School of Global Public Health, The University of North Carolina at Chapel Hill, Chapel Hill, North Carolina, United States of America; 2 Carolina Population Center at The University of North Carolina at Chapel Hill, Chapel Hill, North Carolina, United States of America; 3 The Palladium Group, Chapel Hill, North Carolina, United States of America; London School of Economics, UNITED KINGDOM

## Abstract

**Background:**

Accurate measurement of maternal mortality is needed to develop a greater understanding of the scale of the problem, to increase effectiveness of program planning and targeting, and to track progress. In the absence of good quality vital statistics, interim methods are used to measure maternal mortality. The purpose of this study is to document experience with three community-based interim methods that measure maternal mortality using verbal autopsy.

**Methods:**

This study uses a post-census mortality survey, a sample vital registration with verbal autopsy, and a large-scale household survey to summarize the measures of maternal mortality obtained from these three platforms, compares and contrasts the different methodologies employed, and evaluates strengths and weaknesses of each approach. Included is also a discussion of issues related to death identification and classification, estimating maternal mortality ratios and rates, sample sizes and periodicity of estimates, data quality, and cost.

**Results:**

The sample sizes vary considerably between the three data sources and the number of maternal deaths identified through each platform was small. The proportion of deaths to women of reproductive age that are maternal deaths ranged from 8.8% to 17.3%. The maternal mortality rate was estimable using two of the platforms while obtaining an estimate of the maternal mortality ratio was only possible using one of the platforms. The percentage of maternal deaths due to direct obstetric causes ranged from 45.2% to 80.4%.

**Conclusions:**

This study documents experiences applying standard verbal autopsy methods to estimate maternal mortality and confirms that verbal autopsy is a feasible method for collecting maternal mortality data. None of these interim methods are likely to be suitable for detecting short term changes in mortality due to prohibitive sample size requirements, and thus, comprehensive and continuous civil registration systems to provide high quality vital statistics are essential in the long-term.

## Introduction

An estimated 289,000 women die each year from complications in pregnancy or childbirth with over half of these deaths occurring in sub-Saharan Africa [1]. Millennium Development Goal 5 (MDG5), to improve maternal health, includes a target to reduce the maternal mortality ratio by three quarters by 2015 [2]. As this deadline approaches, awareness about the difficulties in tracking progress toward this goal has been heightened. Accurate measurement of maternal mortality is needed to develop a greater understanding of the scale of the problem, to increase effectiveness of program planning and targeting, and to track progress toward this goal [3]. Without data on deaths and cause of deaths, informed targeting and prioritization of resources based on need is not feasible [4, 5].

There are numerous measurement challenges in estimating reliable maternal mortality statistics. Civil registration systems that track births, deaths and cause of death on a continuous and permanent basis are needed to generate vital statistics about maternal deaths [6, 7]. However, less than 40% of countries have complete civil registration systems with good quality data on cause of death [1]. Resource and capacity constraints hinder collection of death statistics, especially in Africa where the majority of deaths occur outside of health facilities and are not reported and/or certified with information on cause of death. Even where vital statistics registries are in place, underreporting of deaths, incomplete recording at time of death, misclassification of cause of death, and lack of reliable death certification hinder accurate measurement of maternal mortality [8]. Where deaths are registered, the cause of death may not be certified by a physician [9, 6].

In the absence of good quality vital statistics, a variety of interim methods to measure maternal mortality can be used [6, 7]. Population-based interim methods include population census, sample registration systems, demographic surveillance sites, and household surveys [10, 11, 12]. Verbal autopsy is a commonly used approach to identifying causes of death, including maternal deaths, as part of demographic and active surveillance systems, and sample vital registration with verbal autopsy (SAVVY) [12, 13]. Deaths identified through these platforms are followed by a verbal autopsy interview, in which an age-specific questionnaire is administered to the caregiver of the deceased. This interview collects information on the deceased’s history of illness, signs and symptoms of the illness, utilization of health services, and any additional information from available health records from the period leading up to the death. The World Health Organization first established and disseminated standards for verbal autopsy in 2007. These standards included verbal autopsy questionnaires, cause-of-death certification and coding guidelines [14]. Although these standards have been in place for almost a decade and despite the recognized need to conduct research around their use [15], little documentation is available about the experience of implementing these methods, particularly for measuring maternal mortality. The objective of this study is to review and contrast three community-based platforms for measuring maternal mortality using verbal autopsy: (1) a post-census mortality survey (PCMS) in Mozambique, (2) a large-scale demographic household survey (HHS) in Bangladesh, and (3) a sample vital registration system (SAVVY) in Zambia.

## Methods

The first source of data is a post-census mortality survey based on a sample of 10,080 deaths from the 2007 Mozambique census. This survey, known as the Inquiry on Causes of Mortality (INCAM), was the first post-census mortality survey in Africa. The survey used the 2007 World Health Organization (WHO) verbal autopsy tool to classify causes of death consistent with the international classification system (ICD-10). The INCAM sampling frame was the 2007 Mozambique General Census of Population and Housing. The sample was designed to be representative at national, provincial and urban and rural levels. Enumeration areas (EAs) in 10 provinces from the 2007 census were divided into urban and rural strata and then a sample of EAs was randomly selected from each strata. Outside of the capital city the corresponding and adjoining census supervisory areas (CSAs) for each selected EA were combined to form a segment. A total of 144 segments were selected, which were made up of 64 single CSA segments in Maputo City and 80 double CSA segments in the provinces. Census forms from each INCAM segment were reviewed for deaths in the previous 12 months and each death was given a unique identifier to allow for linkage to the subsequent verbal autopsy forms and death certificates.

Of the 18,105 deaths identified through the census in INCAM segments, 9,895 were found and validated, and an additional 185 deaths were identified during the survey. Reasons that deaths initially identified in the census were invalidated include that the death occurred outside of the designated reference period (4,891 deaths), the household was not located (1,562 households), reported decedents resided outside of INCAM enumeration areas, duplicate reporting of deaths, and stillbirths. For the final sample of 10,080 deaths, the age-specific WHO 2007 VA questionnaires were administered with minor adaptations to fit the specific needs of Mozambique (e.g., scorpion added to list of animal bites; list of facilities for treatment adapted to reflect facilities available in Mozambique). Two local physicians reviewed and independently determined a cause(s) of death for each case. Where disagreement occurred in the initial review the physicians worked together to reach consensus on the cause of death [16].

The second source of maternal mortality data is the 2010 Bangladesh Maternal Mortality and Health Care Survey (BMMS-2010). This survey was fielded with 175,621 reproductive-aged women (ages 13–49 years) in 168,629 households and identified maternal deaths that occurred in the selected households since October 2006. The sample was designed to generate nationally representative estimates of maternal mortality with statistical precision to detect a 20 percent decline in MMR (since 2001) with 95 percent significance and 80 percent power. A multi-stage sample selection procedure was used. The primary sampling unit (PSU) for urban areas (formal cities) was ward and for rural and other urban areas was union. In each PSU, two mohallas (urban PSUs) or mouzas (rural and other urban areas) were selected, segmented and a cluster was drawn from each, resulting in 2,708 clusters. Sixty-five households were randomly selected from each cluster for interview. Ninety-eight percent of sampled, occupied households were interviewed. Household deaths were identified through household report. Field work was conducted from January to August 2010. Results on household and maternal deaths presented here are based on deaths in the 36 months before the interview date, excluding the month of interview and refer approximately to the period from early 2007 to early 2010.

Female deaths among women aged 13–49 after the cutoff date of October 2006 were followed up using a verbal autopsy questionnaire to assess cause of death. BMMS-2010 employed a verbal autopsy protocol similar to the verbal autopsy tool designed for BMMS-2001 to maximize comparability with estimates obtained from the 2001 survey. This tool used both structured and unstructured questions that were posed to the most knowledgeable household member regarding the woman’s death. The VA tool was structured differently than the 2007 WHO VA tool. It included separate modules for women who were reported to have died while pregnant but before delivery, for women who were reported to have died during delivery or within 12 months of delivery, and for women who were not pregnant or within a year of delivery at death. The BMMS VA tool includes more detailed questions associated with pregnancy and delivery than the 2007 WHO VA tool for women who died during pregnancy or within 12 months of delivery. The module for women who were not pregnant or within 12 months of delivery at death was more similar to the 2007 WHO VA module than the other two modules. This means that women who were pregnant or within 12 months of delivery at the time of death were asked fewer questions on general symptoms than other women who died or when compared to the 2007 WHO VA tool. To assess cause of death, the completed verbal autopsy was reviewed by two local physicians. For cases unresolved by review and consultation of two physicians, the verbal autopsy was referred to a third local physician for additional review. Deaths that were undetermined after three physician reviews were referred to an expert committee for resolution [17].

The third source of data is a 2009–2010 sample vital registration pilot in Zambia using the SAVVY methodology [18]. The sampling frame used was the 2000 Zambia Census of Population and Housing. Thirty-three CSAs were selected using a one-stage stratified random sample design in four out of Zambia’s nine provinces covering both urban and rural areas. A baseline household census was conducted in January 2010 in the selected CSAs. Deaths in the previous 12-month period were identified from household reports during the census and a verbal autopsy interview was conducted for each death identified. Following the baseline census, key informants were appointed in each community to inform verbal autopsy interviewers of each death that occurred in the CSAs in which they worked on an ongoing basis. Verbal autopsy interviews were conducted for all deaths identified by the key informants. Deaths identified in the 12 months preceding and following the baseline census are included in this analysis, allowing estimation of maternal mortality in the 2009–2010 period in sampled districts.

The 2007 WHO age-specific verbal autopsy tools were used with minor adaptations to the Zambian context, similar to those made for the Mozambique INCAM. Two local physicians reviewed each completed verbal autopsy questionnaire to determine a probable cause of death. In cases of disagreement between the physicians after the initial review, the two physicians reviewed the verbal autopsy together to come to a consensus on the cause of death. Failure to reach a consensus on the cause of death resulted in an “undetermined” cause of death [18].

This study summarizes several key measures of maternal mortality from the three data sources. Maternal death is defined as the death of a woman while pregnant or within 42 days of termination of pregnancy, irrespective of the site or duration of pregnancy, from any cause related to or aggravated by the pregnancy or its management, but not from accidental or incidental causes. We also include late maternal deaths in our analysis, which are defined as the death of a woman from direct or indirect maternal causes more than 42 days but less than one year after the termination of pregnancy [19]. Maternal deaths are further subdivided into direct and indirect causes. Direct causes are maternal deaths resulting from obstetric complications of the pregnancy state (during pregnancy, labor and the puerperium period), from interventions, omissions, incorrect treatment or from a chain of events resulting in any of the above. Indirect causes are maternal deaths resulting from a previous existing disease or disease that developed during pregnancy and which was not due to direct obstetric causes, but which was aggravated by physiologic effects of pregnancy [19]. Using examples from the three case studies, the different platforms and methodologies for conducting verbal autopsies are compared and contrasted, including a discussion of issues related to (1) death identification and classification, (2) estimating maternal mortality ratios and rates, (3) sample sizes and periodicity of estimates, (4) data quality, and (5) cost.

### Ethics statement

This study is a secondary analysis of existing de-identified data sets. As such it was determined to be exempt from institutional ethics review by the Institutional Review Board of the University of North Carolina at Chapel Hill.

## Results

Table 1 provides a comparative summary of selected sample characteristics for the three surveys. The sample sizes vary considerably between the three surveys. The largest total number of deaths identified was in Bangladesh where a three-year recall period was used (18,608 deaths). However, the number of deaths for women of reproductive age (WRA) was greatest in Mozambique with just a one-year recall period (1,643 deaths). Considerably fewer deaths were identified in the Zambia SAVVY pilot, which was based on a much smaller overall sample size. The number of maternal deaths identified through each survey platform was small: 132 maternal deaths in Bangladesh, 259 maternal deaths in Mozambique, and 18 maternal deaths in Zambia.

**Table 1:** Comparison of sample characteristics (unweighted).

	Bangladesh HHS	Mozambique PCMS	Zambia SAVVY
Sample size (# households)	168,629	^a^	17,000
Reference period for deaths	Oct 2006 –interview (Jan–Aug 2010)^b^	Aug 2007 –July 2008	Feb 2009 –Dec 2010^c^
Deaths (#)^d^			
All household deaths	18,608	10,080	1,063
WRA (15–49)	878	1,643	171
Maternal deaths^e^	132	259	18

^a^ The sampling units for the Mozambique survey were deaths identified from the 2007 census not households. The relevant number of households from which deaths were identified is the total number of households in the selected CSA segments, which is unavailable.

^b^ Fieldwork was conducted Jan–Aug 2010. Only deaths 1–36 months before the household interview are included in all subsequent analyses (15,857 household deaths; 768 deaths to WRA; 108 maternal deaths).

^c^ Not all deaths occurring in the latter part of 2010 are expected to be included due to the lag time between a death being identified by a key informant and a verbal autopsy being conducted.

^d^ This table includes all deaths identified. Subsequent tables exclude deaths with missing information on age (0 in Bangladesh, 4 in Mozambique, and 46 in Zambia) or incomplete verbal autopsy data (2 in Bangladesh).

^e^ Maternal death statistics include late maternal deaths (1 in Bangladesh, 46 in Mozambique, 0 in Zambia) and maternal deaths with an underlying cause of HIV/AIDS (0 in Bangladesh, 33 in Mozambique, 3 in Zambia).

Table 2 provides an overview of the cause of death review outcomes for each survey. The percentage of physician reviewers who agreed on the cause of death in the first stage review was slightly lower in Zambia than in the other two surveys. The percentage of deaths among WRA with an undetermined cause of death ranged from 4.1% in Zambia to 10.6% in Bangladesh.

**Table 2:** Verbal autopsy cause of death (COD) review: percent agreement and percent undetermined cause (unweighted).

Survey	Cumulative Reviewer Agreement (%) ^a^				Undetermined COD (%, (n))		
	Stage 1	Stage 2	Stage 3	Stage 4	Total	WRA	Maternal
Bangladesh HHS	78.6	84.8	96.0	100	na	10.6 (768)	8.3 (108)
Mozambique PCMS	74.7	100	NA	NA	6.7 (10,076)	6.9 (1,643)	9.4 (255)
Zambia SAVVY	61.2	100	NA	NA	4.6 (1,063)	4.1 (171)	6.7 (15)

^a^ Stages of review were as follows: (1) review by 2 physicians; (2) consultation between 2 physicians; (3) review by 3^rd^ physician; (4) review by expert committee.

Table 3 provides a summary of maternal mortality estimates by country using each platform. The proportion of deaths to WRA that are maternal deaths in the three countries ranged from 8.8% in Zambia to 17.3% in Mozambique. The maternal mortality rate (MMRate) requires an estimate of the woman-years of exposure among WRA in the reference period for the denominator. This is only available directly from the HHS and SAVVY platforms; for the PCMS, the verbal autopsy survey data has to be linked to the original census data to obtain the denominators. We did not have linked data available to us so were not able to obtain the MMRate for Mozambique. The MMRate for Bangladesh is estimated at 17.0 per 100,000 WRA and in Zambia it is estimated at 69.1 per 100,000 WRA. The maternal mortality ratio (MMR) requires an estimate of the general fertility rate (or number of live births) in the study population in the reference period, which is only available directly from the HHS platform in Bangladesh, providing an estimated MMR of 197 per 100,000 live births.

**Table 3:** Maternal mortality statistics by country and survey platform.^a^

	Bangladesh HH	Mozambique PCMS	Zambia SAVVY
Proportion of deaths that are maternal for WRA (%)	14.2	17.3	8.8
MMRate (per 100,000 women of WRA)	17.0	na	69.1
MMR for WRA (per 100,000 live births)	197	na^b^	na
Type of maternal death			
Direct (%)	63.9	45.2	80.4
Indirect (%)	35.1	37.8	19.6
Late maternal deaths (%)	0.0	17.0	0.0
HIV-related maternal deaths (%)	0.0	19.1	12.9
Maternal deaths (weighted n)	103.8	5,662.5	14.8
Deaths for women 15–49 (weighted n)	732.4	32,733.0	168.3
Exposure (weighted life years)	609,785	na	21,418

^a^ All numbers are weighted unless otherwise specified.

^b^ The INCAM report provides an estimate of the MMR among women age 15–49 of 489.3 per 100,000 live births (Table 32) but this estimate is based on the 2007 census data not on the INCAM data [16].

The percentage of maternal deaths due to direct obstetric causes ranged from 45.2% in Mozambique to 80.4% in Zambia. Seventeen percent of maternal deaths in Mozambique were classified as late maternal deaths. Human Immunodeficiency Virus (HIV) was an underlying cause of death for 19.1% of maternal deaths in Mozambique and 12.9% of maternal deaths in Zambia but was not identified as an underlying cause of death for any maternal deaths in Bangladesh.

**Fig 1:**
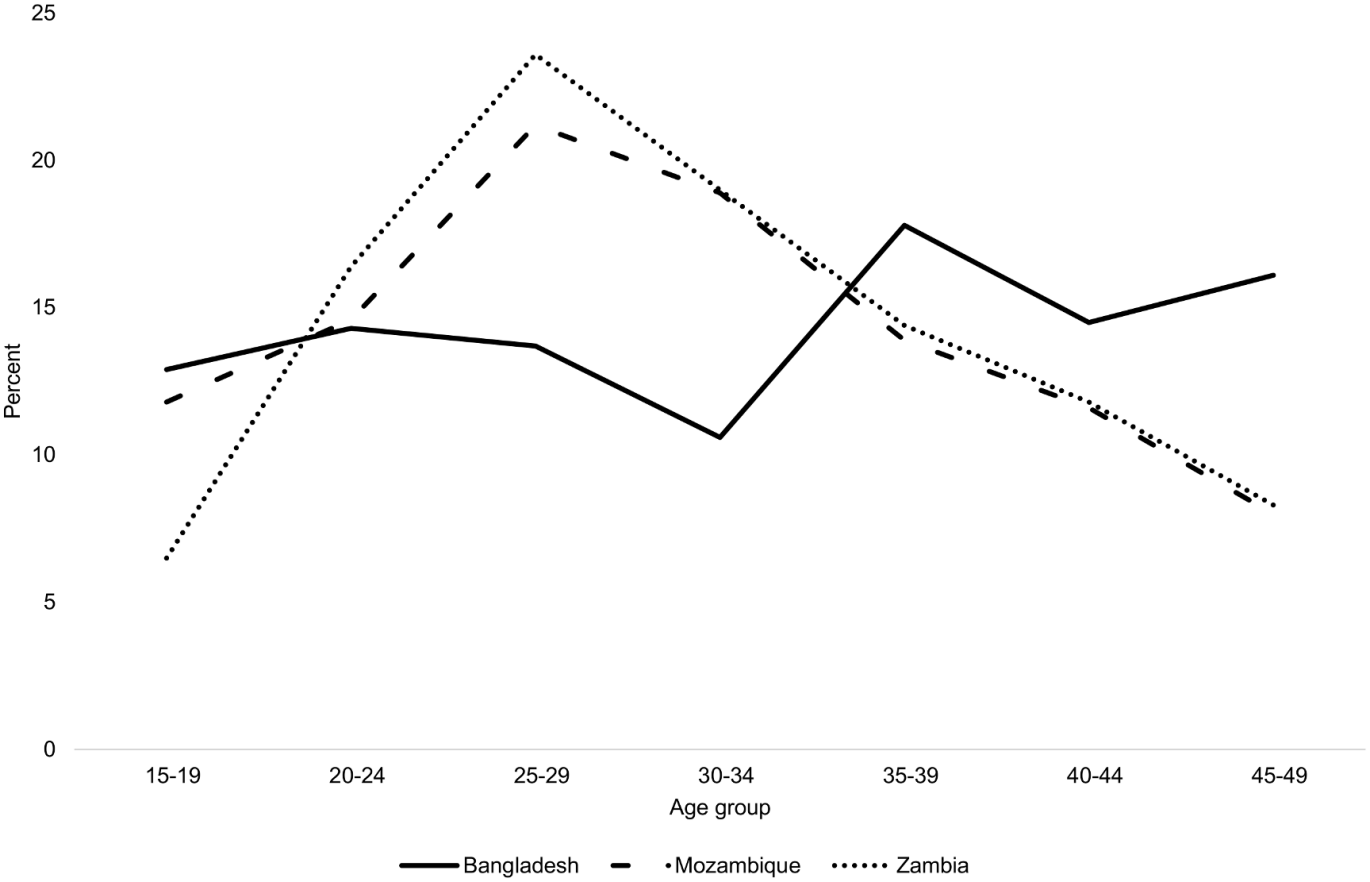
Distribution of deaths among women aged 15–49 by age group and country, weighted.

Table 4 provides a summary of the proportion of deaths by age group for all deaths identified through the survey platforms. Fig 1 presents the proportion of deaths among WRA by age group. Of all deaths, the greatest proportion occur in the under five and 15–49 year age groups in the two African countries, while in Bangladesh, the majority of deaths occur among individuals aged 50 and above. For women of reproductive age, the proportion of deaths peaks at age 25–29 in Mozambique and Zambia and then declines. In Bangladesh the proportion of deaths among WRA increases around age 35.

**Table 4:** Distribution of deaths by age group and country/platform, weighted

Age (years)	Bangladesh HHS	Mozambique PCMS	Zambia SAVVY
All Deaths (men and women)			
Under 5	16.1	42.7	36.0
5–14	2.8	7.4	5.5
15–49	11.7	30.9	36.0
50+	69.5	19.0	22.6
Number	15,315.7	225,047.4	1,066.9

Table 5 provides a snapshot of the share of female deaths that are maternal deaths in each age group for the three survey platforms. The highest percentage of maternal deaths to women of reproductive age was in Mozambique (17.3%); over one-fifth of adult female deaths were maternal deaths in the 15–19 and 20–24 and 25–29 year age groups (26.2%, 26.0%, and 21.7% respectively). In Bangladesh, over one-fifth of deaths among women aged 20–24, 25–29, and 30–34 were maternal (23.9%. 23.4%, and 28.5% respectively). In Zambia, a much smaller proportion of deaths among women are due to maternal causes in each age group. The highest percentage of maternal deaths to WRA in Zambia was 10.6% in the 20–24 year age group. However, the denominators in each age group are small so estimates are subject to large sampling error, particularly in Zambia.

**Table 5:** Maternal deaths as a share of all female deaths by age group (15–49), weighted.

	Bangladesh (HH) % (unweighted n)	Mozambique (PCMS) % (unweighted n)	Zambia (SAVVY) % (unweighted n)
Age (years)			
15–19	7.4 (97)	26.2 (158)	8.2 (12)
20–24	23.9 (108)	26.0 (241)	10.6 (28)
25–29	23.4 (95)	21.7 (345)	10.4 (40)
30–34	28.5 (87)	16.9 (319)	9.6 (31)
35–39	15.8 (138)	11.2 (226)	7.8 (26)
40–44	3.2 (111)	8.2 (198)	9.7 (20)
45–49	1.7 (132)	1.2 (156)	0.0 (14)
**Total**	**14.2 (768)**	**17.3** (1,643)	**8.8 (171)**

**Fig 2:**
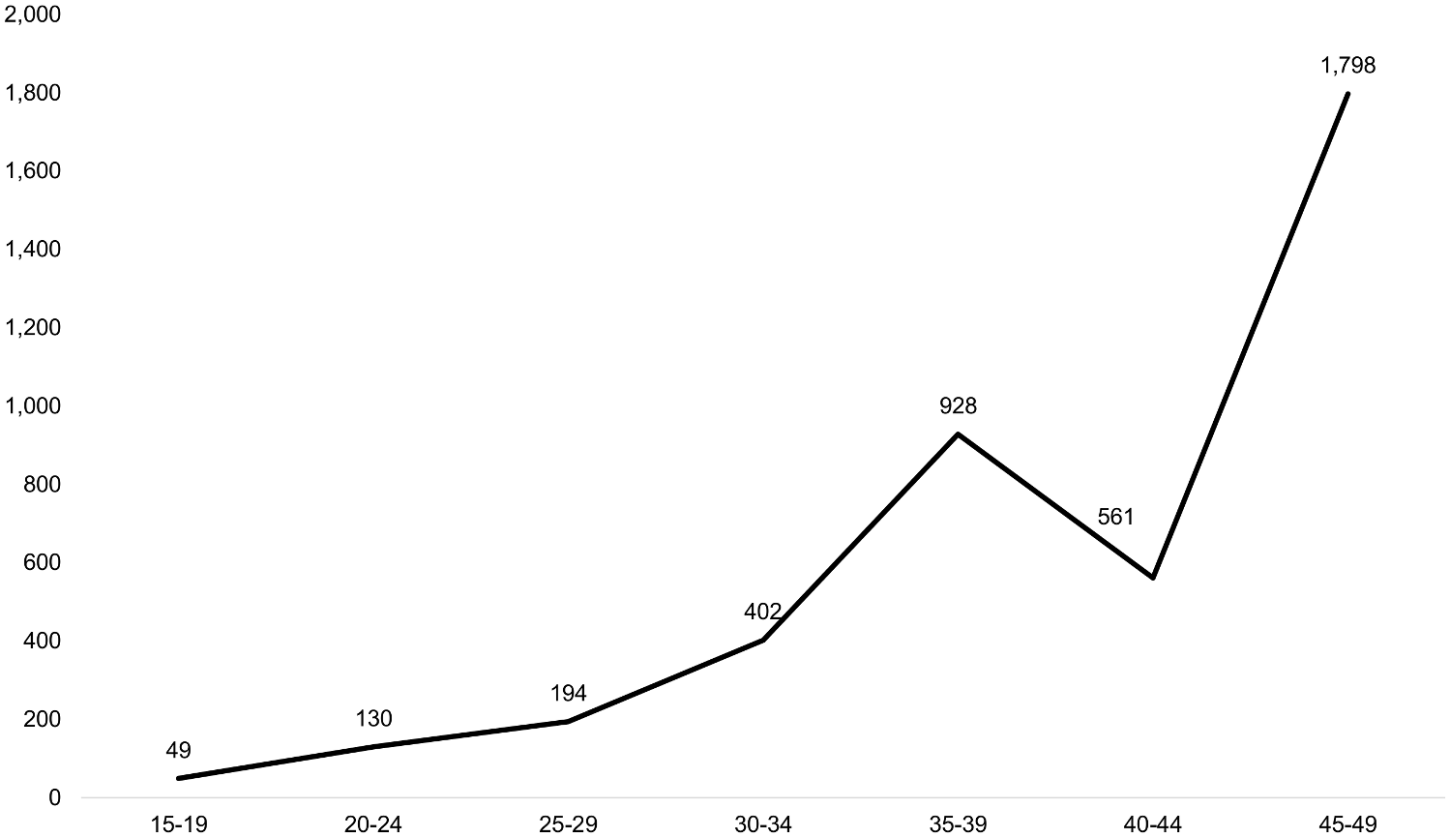
MMR per 100,000 live births by age group, Bangladesh 2010.

One check for the internal consistency of data and maternal death classification is to look at the distribution of the maternal mortality ratio (MMR) by age group. The expected distribution is J-shaped with a relatively high MMR at early ages that dips and then increases again with age, although this pattern varies substantially by country [20, 21]. We are only able to calculate the MMR from the household survey platform data in Bangladesh; Fig 2 presents the MMR by age for that survey. The age pattern of MMR does not exhibit the expected J-shaped pattern but increases with age with the lowest age-specific MMR in the 15–19 year age group.

Table 6 compares the maternal mortality estimates from these three verbal autopsy platforms with estimates from other sources. The estimates from different sources are obtained using different approaches including modeling (WHO and IHME), direct sisterhood method (DHS), and direct estimation from adjusted census data (censuses). DHS and census estimates typically refer to pregnancy-related deaths rather than maternal deaths (direct and indirect). In Bangladesh and Mozambique the BMMS 2010 and INCAM produce higher estimates of the proportion of deaths that are maternal deaths than do the other sources of data. In Zambia, the SAVVY estimate of the proportion of deaths that are maternal is similar to that obtained from other sources. Comparing across countries, the VA platforms examined in this paper suggest potential differences between the three countries in the proportion of deaths that are maternal deaths while the other sources of data suggest that the proportion of deaths that are maternal deaths are quite similar across the three countries. The Maternal Mortality Rate can only be compared across sources for Zambia where the SAVVY estimate of the maternal mortality rate is similar to that obtained using the direct sisterhood method for pregnancy-related deaths in the 2013–14 DHS and the 2010 census, and a bit lower than the estimate obtained from the 2007 DHS (although likely within sampling error). The MMR from the BMMS-2010 is similar to recent estimates from WHO (2013) and the census (2011).

**Table 6:** Comparison of Maternal Mortality Estimates: Zambia, Bangladesh, Mozambique.

	MMR	MMRate	Proportion Maternal
**Bangladesh**			
*BMMS 2010* ^a^	*197*	*17*	*14*.*2*
WHO 2013^b^	170	NR	7.6
HME 2011^c^	247	NR	NR
WHO 2010^d^	240	NR	5.7
Census 2011^e^	218		9.5
**Mozambique**			
*INCAM 2008* ^f^	*-*	*-*	*17*.*3*
WHO 2013^b^	480	NR	8.2
DHS 2011^g^	408	76	13.9
IHME 2011^c^	510	NR	NR
WHO 2010^d^	490	NR	7.7
Census 2007^f^	489	NA	NA
**Zambia**			
*SAVVY 2009–201* ^h^	*-*	*69*.*1*	*8*.*8*
DHS 2013–2014^i^	398	74	9.5
WHO 2013^b^	280	NR	7.9
SMGL 2011–2012^j^	505	101	13.3
IHME 2011^c^	293	NR	NR
Census 2010^j^	483	86	9.3
WHO 2010^d^	440	NR	9.1
DHS 2007^k^	591	117	8.9

Sources:

^a^ National Institute for Population Research and Training, MEASURE Evaluation, International Centre for Diarrhoeal Disease Research (2012) Bangladesh Maternal Mortality and Health Care Survey 2010. Available: http://www.cpc.unc.edu/measure/publications/tr-12-87. Accessed October 15, 2012.

^b^ World Health Organization (ND) WHO Maternal Mortality Country Profiles. Available: www.who.int/gho/maternal_health/en/#M. Accessed 1 March 2015.

^c^ Lozano R, Wang H, Foreman KJ, Rajaratnam JK, Naghavi M, Marcus JR, et al. (2011) Progress towards Millennium Development Goals 4 and 5 on maternal and child mortality: an updated systematic analysis. Lancet 378(9797): 1139–65. 10.1016/S0140-6736(11)61337-8

^d^ UNFPA, UNICEF, WHO, World Bank (2012) Trends in maternal mortality: 1990–2010. Available: http://www.unfpa.org/public/home/publications/pid/10728. Accessed 7 October 2012.

^e^ Bangladesh Bureau of Statistics, Statistics Informatics Division, Ministry of Planning (December 2012) Population and Housing Census 2011, Socio-economic and Demographic Report, National Series–Volume 4. Available at: http://203.112.218.66/WebTestApplication/userfiles/Image/BBS/Socio_Economic.pdf. Accessed 15 February, 2015.

^f^ Mozambique National Institute of Statistics, U.S. Census Bureau, MEASURE Evaluation, U.S. Centers for Disease Control and Prevention (2012) Mortality in Mozambique: Results from a 2007–2008 Post-Census Mortality Survey. Available: http://www.cpc.unc.edu/measure/publications/tr-11-83. Accessed 6 October 2012.

^g^ Ministerio da Saude (MISAU), Instituto Nacional de Estatística (INE) e ICF International (ICFI). Moçambique Inquérito Demográfico e de Saúde 2011. Calverton, Maryland, USA: MISAU, INE e ICFI.

^h^ Mudenda SS, Kamocha S, Mswia R, Conkling M, Sikanyiti P, et al. (2011) Feasibility of using a World Health Organization-standard methodology for Sample Vital Registration with Verbal Autopsy (SAVVY) to report leading causes of death in Zambia: results of a pilot in four provinces, 2010. Popul Health Metr 9:40. 10.1186/1478-7954-9-40

^i^ Central Statistical Office (CSO), Ministry of Health (MOH), Tropical Diseases Research Centre (TDRC), University Teaching Hospital Virology Laboratory, University of Zambia, and ICF International Inc. 2014. Zambia Demographic and Health Survey 2013–14: Preliminary Report. Rockville, Maryland, USA. Available: http://dhsprogram.com/pubs/pdf/PR53/PR53.pdf. Accessed February 26, 2015.

^j^ Centers for Disease Control and Prevention (2014) Saving Mothers, Giving Life: Maternal Mortality.

Phase 1 Monitoring and Evaluation Report. Atlanta, GA: Centers for Disease Control and Prevention, US Dept of Health and Human Services. Available at: http://www.savingmothersgivinglife.org/doc/Maternal%20Mortality%20(advance%20copy).pdf. Accessed 26 February 2015.

^k^ Central Statistical Office (CSO), Ministry of Health (MOH), Tropical Diseases Research Centre (TDRC), University of Zambia, and Macro International Inc. 2009. Zambia Demographic and Health Survey 2007. Calverton, Maryland, USA: CSO and Macro International Inc.

## Discussion

This study documents recent experiences applying standard verbal autopsy methods to estimate maternal mortality. It confirms that verbal autopsy is a feasible method for collecting maternal mortality data in the absence of reliable vital registration data. The comparison of different platforms for conducting verbal autopsy (e.g., sample vital registration, post-census mortality survey, and stand-alone household survey) provides insights for those considering applying verbal autopsy methods to estimate levels and trends in maternal mortality. Following is a discussion of key issues identified through this study with a summary provided in Table 7 by survey platform.

**Table 7:** Summary of the advantages and disadvantages of different verbal autopsy platforms.

Survey Platform: Post census mortality survey in Mozambique
Deaths identification method: Deaths identified in selected census enumeration areas and validated through PCMS
Advantages
• Cost of death identification is absorbed by census
• May increase targeted sample size easily by adjusting sampling fraction
• 12 month recall period is standard on most censuses, accepted as ‘reasonable’ by many verbal autopsy practitioners
• Ability to calculate cause-specific mortality fractions at subnational level
• May be able to leverage multi-donor/sectoral financial support
• Data quality can be checked by comparing mortality information with the (overall) mortality statistics produced by the census
Disadvantages
• Since PCMS builds on census, can only be conducted every 10 years
• Census data quality may be relatively poorer quality; many out-of-frame deaths identified in census by PCMS
• Requires link back to census data to calculate rates and ratios
• Requires long lead time for planning (estimated 15+ months before census)
• Requires 2 visits to household, first to identify the death and then follow-up for the verbal autopsy; may result in loss of HHs that cannot be re-identified a second time
Survey Platform: Household survey in Bangladesh
Death identification method: Deaths identified and validated by household questionnaire
Advantages
• Fieldwork is logistically relatively simple, can be planned for in 4–6 months
• Allows for flexibility in terms of timing of verbal autopsy (during initial HH visit/survey or at follow-up) and duration of recall period
• 3-year recall period allowed for more cost-effective identification of deaths
Disadvantages
• Sample size of deaths relatively small (~900 female deaths, 15–49) even with large sample size
• Concerns in the literature that the use of a 3- year recall period may yield uncertain verbal autopsy data quality
• Can only detect large relative changes in MMR, which limits the frequency with which surveys can be repeated
Survey Platform: SAVVY
Death identification method: Deaths identified through sample vital registration in selected Census Supervisory Areas
Advantages
• Allows for flexibility in terms of timing of verbal autopsy (during initial HH visit or at follow-up)
• Continuous data collection is advantage once the system is up and running
Disadvantages
• Although it provides ongoing data, sample size per year is likely to be too small to detect short term change; need to build up a sample of deaths over time.

### Death identification and classification

Accurate recall of deaths is a concern with any of the platforms, as both under-reporting and telescoping of deaths is a known concern with self-reported data[11, 22]. Identifying deaths from the census for the post-census mortality survey resulted in a large number of deaths that were outside of the 12-month reference period. This problem has been noted in other post-census survey efforts to obtain data on maternal mortality [23]. Hakkert (2011) also noted other problems with post-census surveys to estimate maternal mortality including omission of deaths, and misclassification of deaths as pregnancy-related in the census that were found not to be pregnancy-related at follow up, and vice-versa. Follow-up validation of deaths identified through the census can help to mitigate forward telescoping, as was done in PCMS, but does not address underreporting. The SAVVY platform in Zambia is the only one to collect data on deaths prospectively (but relies on accurate reporting by community key informants), although in this paper we also used recalled data for the 12 months prior to the baseline census.

Recall of the specific circumstances leading to death may not be accurate, and longer recall periods may exacerbate this problem. The highest percentage of undetermined causes of death for WRA was observed in Bangladesh despite having the most rigorous cause of death assignment process. Bangladesh used a three-year recall period for the verbal autopsy whereas the other two platforms used a one-year period. Verbal autopsy field practitioners do not generally recommend using verbal autopsy methodologies beyond one year of recall [24]. However, a review of the Bangladesh data did not find any evidence that undetermined cause of death was more common for deaths earlier in the 3-year recall period (data not shown). There was some suggestion that undetermined cause of death may have been more common for interviews conducted earlier in fieldwork suggesting that the quality of the VA interviews improved as interviewers got more experienced, but numbers are too small to draw definitive conclusions. The VA instrument used in Bangladesh also deviated somewhat from the 2007 WHO standard VA tool and collected less detail on non-maternal causes of death, particularly for women who were pregnant or within a year of delivery at the time of death. Overall, however, the quality of death identification and basic demographic data for the BMMS using a 3-year recall period appears to be good.

The level of agreement during physicians review ranged between 61–79% in the first stage for the three surveys, but this level improved with consensus review (either involving a third coder, or two coders reviewing together the discrepant VAs). The potential for human bias in coding of completed VA questionnaires is a concern with physician coded VA [15]. Additionally, physician-coded VA requires considerable time and has cost implications. Since the implementation of the three studies covered in this analysis, automated methods for coding cause of death from verbal autopsies have become more common and more recent revisions of the WHO standard verbal autopsy tools are designed to work better with publically available software for assigning cause of death [25]. The 2007 WHO VA tools were designed for use with physician coding, however, applying automated coding methods such as the InterVA with these data would require some assumptions to be made to conform to the interVA requirements, and there is not yet strong evidence supporting the use of automated methods over physician-coded methods [26]. Future applications of VA to assess maternal mortality should explore use of these coding methods as an alternative to physician coding, particularly if they use the more recent WHO standard VA tools.

### Estimating maternal mortality ratios and rates

The different platforms have different implications for estimation of common maternal mortality indicators. With the data available to us we were able to estimate the proportion of deaths that were maternal deaths from all three platforms. However, only the household survey platform dataset in Bangladesh had all the data needed to calculate both the MMRate and the MMR. The PCMS platform in Mozambique did not permit calculation of the MMRate or the MMR without further linking back to census data to obtain the relevant denominators. The ability to link the verbal autopsy data to the original census data in a timely way and make linked data available for analysis is an important consideration for the use of a PCMS platform for estimation of maternal mortality indicators. Getting access to national census data to allow for timely linkage with mortality data can be a lengthy process and hence limit some analyses that require use of census data. The SAVVY pilot data in Zambia permitted calculation of the MMRate but the lack of data on live births prevented calculation of the MMR. Data on live births is now being collected in the scale up of SAVVY in Zambia to address this limitation so future data should allow calculation of the MMR. With all platforms, the data collection instruments need to anticipate analysis needs and include data on relevant denominators that can be linked to the verbal autopsy data.

In principle, the proportion of deaths that are maternal deaths, which was available from all three platforms, could be applied to other existing data on the age-sex distribution of the population and of deaths and on the General Fertility Rate to obtain estimates of MMRate and MMR. In practice age-sex distributions of deaths are not always readily available and adjustment to the raw data are often needed as different sources of data are likely to be subject to different types and magnitudes of error. The modeling methods used by the WHO and IHME to obtain estimates of maternal mortality use the proportion of deaths that are maternal deaths from sources such as the INCAM in Mozambique and the BMMS-2010 as inputs to their models but adjust the raw data prior to using it in the model [27, 1].

### Sample sizes and periodicity of estimates

The Zambia SAVVY data were from a pilot covering 17,000 households and only 18 maternal deaths were identified over almost 2 years. Even with relatively large sample sizes (over 168,000 households in Bangladesh and census data from 224 CSAs in Mozambique), relatively small numbers of maternal deaths were identified. Hakkert (2011) noted that because the Mozambique INCAM was designed to collect data on all causes of death not just maternal deaths, the sample size was insufficient for precise estimation of maternal mortality [23]. The scale up of the Zambia SAVVY will cover approximately 34,600 households so, assuming the cause specific mortality fractions and MMRates are within the range observed in the pilot, relatively small numbers of maternal deaths per year could be expected there as well. The Zambia SAVVY is also designed to produce mortality estimates for all causes of deaths not just maternal mortality.

These numbers have implications for the ability of any of these platforms to provide estimates of change in maternal mortality over short periods of time. Small fluctuations in the numbers of death represent large relative changes in the mortality rate. One potential drawback of the PCMS platform is that it can only be used every 10 years or so when a census is conducted, limiting the frequency of estimates. However, the other platforms are also likely to only be able to estimate changes over relatively long periods of time given cost and logistical limitations to sample sizes and associated statistical precision. The SAVVY platform has the advantage of providing data on a continuous basis but small variations in the number of maternal deaths per year will translate into large relative variation in the rates observed so data will need to be cumulated over several years to obtain stable estimates. Although the SAVVY platform in Zambia is only intended to provide the levels and patterns of all-cause mortality at the national level, SAVVY tools and methodology may be adapted to estimate cause-specific mortality, such as maternal mortality, by increasing the sample size so that it is large enough for meaningful analysis and interpretation. For example, SAVVY has been adapted as an evaluation tool for the Saving Mothers Giving Life Initiative in Zambia, whereby the baseline survey conducted a complete enumeration of WRA in selected districts. For other diseases with high prevalence in Zambia such as malaria and HIV, the number of deaths from such causes in the SAVVY sample is large enough to detect changes over time within a relatively short time interval. Sample size challenges will increase as mortality and fertility rates decline.

### Data quality

Estimates obtained through the use of verbal autopsy with these survey platforms appear plausible and are generally comparable to statistics obtained through other surveys and methodologies, except that the proportion of deaths of women of reproductive age that were classified as maternal deaths is somewhat higher in the Bangladesh survey and the Mozambique PCMS than obtained from modeled estimates. This could suggest that deaths are over-classified as maternal deaths in these platforms, or that non-maternal deaths are underestimated, or that the modeled estimates are too low. The MMR in Bangladesh is comparable to other recent sources however. Several of the maternal deaths in our sample were missing information about age at death, which resulted in their exclusion from age-related analyses. Since maternal death is such a rare event, exclusion of even just a few deaths can have an impact on the results. In cases where a respondent may not know the exact age of the deceased at time of death, gathering information on estimated age at death, for example in a five- or ten-year ranges, or imputation of age at death could be useful.

Analysis of BMMS data indicates that data were missing for below 0.1% of cases [17]. However, distortions exist with regard to heaping of reported age for current household members and household deaths for years ending in zero and five, and are more pronounced for females than males. This imprecise reporting, although not uncommon, may be associated with age exaggeration, and in the case of our 5-year age analyses, may shift the distribution of maternal deaths to the right.

Overall, age patterns in maternal deaths are broadly consistent with expectations, with some exceptions. In the two African countries, which experience relatively high fertility and higher levels of overall mortality, deaths among women of reproductive age peak among women age 25–29, which coincides with the prime ages for childbearing. This pattern is consistent with the distribution of maternal deaths by age observed in multi-country analysis [20, 21]. In Bangladesh the age distribution of deaths among women of reproductive age does not follow this pattern. This is likely associated with the lower fertility and mortality rates in Bangladesh and its different age structure. Maternal deaths represent a greater share of all deaths among women under 30 in Mozambique and among women age 20–34 in Bangladesh, coinciding with higher fertility ages. This pattern is not seen as clearly in the Zambia data; however the numbers in each age group are too small to conclude much from this observation. We were only able to look at the age pattern in the MMR in Bangladesh where it did not conform to the expected J-shaped pattern. The MMR did rise steeply at older ages but was not higher among 15–19 years olds who actually experienced the lowest MMR. While not expected, similar patterns have been documented in other countries and recent evidence suggests the expected excess risk among adolescents may have been over-stated [20, 21, 28].

### Cost considerations

Because maternal death is a relatively rare event and its measurement requires large sample sizes, the costs of data collection to estimate maternal mortality are high. Costs include not only those for the data collection itself, but also for pilot testing, training, field supplies and equipment, technical assistance, data processing and analysis, and other miscellaneous expenses. Each verbal autopsy platform has different implications for costing and each platform has different advantages and disadvantages. For example, using a PCMS platform may save cost in terms of death identification but data must also be linked back to the original census to obtain the MMRate and MMR, and the number of out of scope deaths identified is a concern and requires rigorous validation, each of which has its own cost implications. Implementing a stand-alone survey such as the BMMS is costly, but allows for flexibility and oversight and, if designed correctly, allows for calculation of all maternal mortality indicators and collection of additional background information (e.g. on use of maternal health services). However, unlike the other two platforms, the BMMS did not allow calculation of mortality rates and cause of death distributions for men or for women under 15 or 50 and over. A SAVVY platform provides continuous data allowing data to be built up over time but requires sustained funding for supervision and training of staff on an ongoing basis.

### Limitations

Although this study is primarily descriptive, it has provided a number of insights regarding the use of verbal autopsy with three different platforms to estimate maternal mortality indicators. We are unable to compare these estimates with a “gold standard” value such as data from complete vital statistics registries. However, the lack of complete vital registration is the very reason that verbal autopsy methods are needed to estimate cause-specific mortality. Evaluating several components of data quality provide reassurance that the verbal autopsy tool provides reasonably reliable information. Several limitations exist related to the source data that have been described, including lack of denominator data required for calculating maternal mortality rates and ratios such as the number of women of reproductive age and live births in the surveyed areas. These data limitations restrict the extent of external comparisons that are possible in this analysis.

## Conclusions

These and other similar community-based methods are interim measures that fill the gap for accurate statistics on mortality and its causes. Hence, they are complements, rather than substitutes, to other facility-based information systems (e.g., maternal health information systems within the health sector) and a fully functioning vital registration system. This study demonstrates that all of these platforms are viable options for collecting maternal mortality estimates with verbal autopsy, with some caveats, particularly for the census platform. However, none of these interim methods are likely to be suitable for detecting short term changes in mortality due to prohibitive sample size requirements. Although interim methods can and do provide useful estimates of maternal mortality, in practice, it may be optimal to use a range of methods to triangulate best estimates of maternal mortality until comprehensive and continuous civil registration systems that provide high quality vital statistics are available in the long-term. However, reconciliation of different estimates from different sources needs to be considered to avoid confusion among data users, and using multiple methods has non-trivial cost implications. Choice of an appropriate interim method should be tailored to the statistical strengths and weaknesses of each method available, as well as the local context (e.g., existing vital statistics infrastructure, budgetary considerations, human resource capacity, timing, political commitment, a legal framework, and public trust) [7, 12].

## References

[1] WHO, UNICEF, UNFPA, The World Bank, United Nations Population Division. Trends in maternal mortality: 1990 to 2013. Estimates by WHO, UNICEF, UNFPA, The World Bank and the United Nations Population Division. 2014. Available: http://apps.who.int/iris/bitstream/10665/112682/2/9789241507226_eng.pdf?ua=1.

[2] United Nations. End poverty 2015. Goal 5: Improve maternal health. ND. Available: http://www.un.org/millenniumgoals/maternal.shtml.

[3] UNFPA, UNICEF, WHO, World Bank. Trends in maternal mortality: 1990–2010. 2012. Available: http://www.unfpa.org/public/home/publications/pid/10728.

[4] Okonjo-Iweala N, Osafo-Kwaako P. Improving health statistics in Africa. Lancet. 2007; 370: 1527–1528. 17980722 10.1016/S0140-6736(07)61644-4

[5] Shibuya K, Scheele S, Boerma T. Health statistics: time to get serious. Bull World Health Organ. 2005; 83(10): 722 S0042-96862005001000002 16283042 PMC2626417

[6] Setel PW, Macfarlane SB, Szreter S, Mikkelsen L, Jha P, Stout S, et al A scandal of invisibility: Making everyone count by counting everyone. Lancet. 2007; 370: 1569–1577. 17992727 10.1016/S0140-6736(07)61307-5

[7] AbouZahr C, Cleland J, Coullare F, Macfarlane SB, Notzon FC, Setel P, et al The way forward. Lancet. 2007; 370: 1791–1799. 18029003 10.1016/S0140-6736(07)61310-5

[8] Say L, Chou D. Better understanding of maternal deaths—the new WHO cause classification system. BJOG. 2011; 118 (Suppl. 2): 15–17. 10.1111/j.1471-0528.2011.03105.x 21951496

[9] Mathers CD, Fat DM, Inoue M, Rao C, Lopez AD. Counting the dead and what they died from: An assessment of the global status of cause of death data. Bull World Health Organ. 2005; 83(3): 171–177. 15798840 PMC2624200

[10] Hill K, Lopez AD, Shibuya K, Jha P. Interim measures for meeting needs for health sector data: Births, deaths, and causes of death. Lancet. 2007; 370: 1726–1735. 18029005 10.1016/S0140-6736(07)61309-9

[11] Stanton C, Hobcraft J, Hill K, Kodjogbe N, Mapeta WT, Munene F, et al Every death counts: Measurement of maternal mortality via a census. Bull World Health Organ. 2001; 79(7): 657–664. 11477969 PMC2566460

[12] Graham WJ, Foster LB, Davidson L, Hauke E, Campbell OMR. Measuring progress in reducing maternal mortality. Best Pract Res Clin Obstet Gynaecol. 2008; 22: 425–445. 10.1016/j.bpobgyn.2007.12.001 18308640

[13] Singh K, Hart L. A Guide on Conducting a Post-Census Verbal Autopsy to Estimate Maternal Mortality. 2015. Available: http://www.cpc.unc.edu/measure/publications/ms-15-102.

[14] World Health Organization. Verbal Autopsy Standards: Ascertaining and attributing causes of death. 2007. Available: http://www.who.int/healthinfo/statistics/verbalautopsystandards/en/index1.html.

[15] Baiden F, Bawah A, Biai S, Binka F, Boerma T, Byass P, et al Setting international standards for verbal autopsy. Bull World Health Organ. 2007; 85(8): 570–1. 17768508 10.2471/BLT.07.043745 PMC2636382

[16] Mozambique National Institute of Statistics, U.S. Census Bureau, MEASURE Evaluation, U.S. Centers for Disease Control and Prevention. Mortality in Mozambique: Results from a 2007–2008 Post-Census Mortality Survey. 2012. Available: http://www.cpc.unc.edu/measure/publications/tr-11-83.

[17] National Institute for Population Research and Training, MEASURE Evaluation, International Centre for Diarrhoeal Disease Research. Bangladesh Maternal Mortality and Health Care Survey 2010. 2012. Available: http://www.cpc.unc.edu/measure/publications/tr-12-87.

[18] Mudenda SS, Kamocha S, Mswia R, Conkling M, Sikanyiti P, Potter D, et al Feasibility of using a World Health Organization-standard methodology for Sample Vital Registration with Verbal Autopsy (SAVVY) to report leading causes of death in Zambia: results of a pilot in four provinces, 2010. Popul Health Metr. 2011; 9:40 10.1186/1478-7954-9-40 21819583 PMC3160933

[19] World Health Organization. Statistical Classification of Diseases and Related Problems. 10th revision Geneva, Switzerland; 1992.

[20] Blanc AK, Winfrey W, Ross J. New findings for maternal mortality age patterns: aggregated results for 38 countries. PLOS ONE. 2013; 8(4): e59864 10.1371/journal.pone.0059864 23613716 PMC3629034

[21] Nove A, Matthews Z, Neal S, Camacho AV. Maternal mortality in adolescents compared with women of other ages: evidence from 144 countries. The Lancet Global Health. 2014; 2(3): e155–e164. 10.1016/S2214-109X(13)70179-7 25102848

[22] Gaskell GD, Wright DB, O'Muircheartaigh CA. Telescoping of landmark events: Implications for survey research. Public Opin Q. 2000; 64(1): 77–89. 10810077 10.1086/316761

[23] Hakkert R. Follow-up surveys for census estimates of maternal mortality: experiences from Bolivia and Mozambique. J Pop Research. 2011; 28:15–30.

[24] Soleman N, Chandramohan D, Shibuya K. Verbal autopsy: current practices and challenges. Bull World Health Organ. 2006; 84: 239–245. 16583084 10.2471/blt.05.027003 PMC2627297

[25] World Health Organization. Verbal autopsy standards: ascertaining and attributing causes of death. 2014. Available: http://www.who.int/healthinfo/statistics/verbalautopsystandards/en/.

[26] Leitao J, Desai N, Aleksandrowicz L, Byass P, Miasnikof P, Tollman S, et al Comparison of physician-certified verbal autopsy with computer-coded verbal autopsy for cause of death assignment in hospitalized patients in low- and middle-income countries: systematic review. BMC Medicine. 2014; 12:22 10.1186/1741-7015-12-22 24495312 PMC3912516

[27] Lozano R, Wang H, Foreman KJ, Rajaratnam JK, Naghavi M, Marcus JR, et al Progress towards Millennium Development Goals 4 and 5 on maternal and child mortality: an updated systematic analysis. Lancet. 2011; 378(9797): 1139–65. 10.1016/S0140-6736(11)61337-8 21937100

[28] Blanc AK. Excess risk of maternal mortality in adolescent mothers. The Lancet Global Health. 2014; 2(4): e201 10.1016/S2214-109X(14)70028-2 25103056

